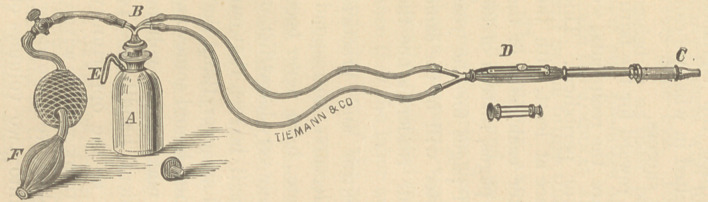# Editorial Notices

**Published:** 1877-06

**Authors:** 


### Editorial Notices.

The index to this volume will appear with the next number.

---

Owing to the report of the convention of the Illinois State Medical Society, reports from other societies and other matters of interest could not be published in this issue.

---

### A NEW THERMO-CAUTERY.

An ingenious and novel device by which thermo-cauterization may be produced, has recently been perfected by Dr. Guérard. We append a cuts howing the instrument. One or two ounces of benzine are put into the bottle (*a*), which is then closed with the rubber stopper (*b*), when the cautery point is selected and screwed to the front of the instrument (*c*). The guard is drawn open with the thumb-button (*d*), and the bottle hooked to a button-hole in the coat by the hook (*e*). The hand-ball (*f*) is worked gently with the left hand, the instrument being held in the right with its point over a lighted lamp which will ignite the gas. When there is a good blast, close the guard by pushing the thumb-button (*d*) forward, and the platinum cauter may be heated to the required intensity and kept so by occasioned compression of the hand-ball.

Our readers will remember that the American Medical Association will meet in Chicago on June 5th.

---

Prospect.—When, a few months since, we were cast upon our own resources for the publication as well as the editorial management of the Journal and Examiner, we were not without apprehension as to the difficulties we should be called upon to encounter, and our ability to surmount them. Unaccustomed to the transaction, in detail, of all the business necessary for the pecuniary success of so important an enterprise, we could not help looking into the future with some anxiety. This solicitude, however, prompted the vigilance, industry and forethought which have resulted in what we can, without hesitancy, proclaim a triumphant success.

As evidence of the latter fact, and as a reward for the faithful and liberal patronage of which we are the recipients, we propose adding sixteen more pages to our periodical. This will place us on a par, in respect to space at least, with the best American medical journals. Hereafter each issue of the Journal and Examiner will contain 112 pages, instead of 96, as heretofore. The aggregate number of pages for the year will therefore be 1,344. The subject-matter will be the same in variety and value as we have given our readers since the establishment of our joint periodical.

Our arrangements for obtaining scientific and practical contributions, hospital reports, clinical lectures, correspondence, local and general news items, etc., etc., have never been so good as they are now. Add to this our very large list of American and foreign exchanges, and it will be seen that our facilities for making an *excellent* journal are everything that could be desired. Already large, and when bound somewhat bulky; with the additions we are now making, our volume will become too ponderous for convenient handling. To remedy this inconvenience, and to afford us the opportunity for adopting other conditions which we think will be advantageous to our readers, as well as ourselves, we have determined to divide the yearly publication into two volumes of six months each; the one to begin in January, and the other in July. This number will therefore terminate the present volume, and will accordingly be furnished with an index. The completion of the volumes, semi-annually, will enable those who desire to do so, to commence their subscription in January and July, and still obtain a complete volume.

The yearly subscription price will remain the same, if paid in advance, according to our published terms. We cannot point out to those who patronize medical periodicals how they can get more substantial reading for the same expenditure.

We take this occasion to express our warmest thanks to the many friends who have stood by us so faithfully during the years gone bye. To our new friends who have recently extended to us their patronage we say welcome; to both we renew our promises of industrious and unremitting efforts to make the Journal and Examiner more worthy of their good will in the future than it has been in the past.

The Journal and Examiner has now nearly completed its second year, and we think it no exhibition of vain affection for our favorite charge, to say that with each issue it has shown increased vigor and comeliness; and that we are continually presented with facts which give us gratifying assurance that the labor and pains taken with it receive the hearty approbation of the profession wherever it is read.

Apology.—We owe it to our readers and to ourselves, to state that a notice announcing the meeting of the Illinois State Medical Society, held in Chicago last month, was to appear in our last issue; but the printers, when arranging the forms, inadvertently left it out. We were greatly chagrined at this unfortunate accident, but felt considerably relieved when we saw that the convention was very well attended nevertheless.

## Figures and Tables

**Figure f1:**